# 3D human bone marrow stromal and endothelial cell spheres promote bone healing in an osteogenic niche

**DOI:** 10.1096/fj.201801114R

**Published:** 2018-11-07

**Authors:** Stefanie Inglis, Janos M. Kanczler, Richard O. C. Oreffo

**Affiliations:** Bone and Joint Research Group, Centre for Human Development, Stem Cells, and Regeneration, Institute of Developmental Sciences, University of Southampton, Southampton General Hospital, Southampton, United Kingdom

**Keywords:** coculture, bone drill defect, cell construct, organotypic culture, *ex vivo*

## Abstract

The current study used an *ex vivo* [embryonic day (E)18] chick femur defect model to examine the bone regenerative capacity of implanted 3-dimensional (3D) skeletal–endothelial cell constructs. Human bone marrow stromal cell (HBMSC) and HUVEC spheroids were implanted within a bone defect site to determine the osteogenic potential of the skeletal–endothelial cell unit. Cells were pelleted as co- or monocell spheroids and placed within 1-mm-drill defects in the mid-diaphysis of E18 chick femurs and cultured organotypically for 10 d. Micro-computed tomography analysis revealed significantly (*P* = 0.0001) increased levels of bone volume (BV) and BV/tissue volume ratio in all cell-pellet groups compared with the sham defect group. The highest increase was seen in BV in femurs containing the HUVEC and HBMSC monocell constructs. Type II collagen expression was particularly pronounced within the cell spheres containing HBMSCs and HUVECs, and CD31-positive cell clusters were prominent within HUVEC-implanted defects. These studies demonstrate the importance of the 3D osteogenic-endothelial niche interaction in bone regeneration. Elucidating the component cell interactions in the osteogenic-vascular niche and the role of exogenous factors in driving these osteogenic processes will aid the development of better bone reparative strategies.—Inglis, S., Kanczler, J. M., Oreffo, R. O. C. 3D human bone marrow stromal and endothelial cell spheres promote bone healing in an osteogenic niche.

The combined actions of cartilage and preosteogenic and vascular cell components are essential in the promotion of endochondral bone healing (1–4). Previous studies have drawn on osteogenesis during development, including cues for cell commitment, osteogenic and chondrogenic cell arrangement, and differentiation driven by osteoid secretion as well as the key role of the vasculature in the patterning of bone, to improve bone tissue engineering strategies (5, 6). Equally important are the underlying cellular processes and interactions of bone maintenance that exist within the bone marrow microenvironment of stem and progenitor cells; this cell niche interaction is crucial for cell proliferation, migration, differentiation, and survival (7, 8).

Over the last 20 years, 3-dimensional (3D) spheroid models have been recognized by cell biologists and tissue engineers as an important tool to study cell-cell interaction together with the extracellular matrix created in these spheroids (9–14). The cell-to-cell contact within pellet cultures is dictated by signaling proteins, including integrins to stimulate the secretion of extracellular matrix that can aid the natural growth and movement of cells (9, 15–17). We have previously shown that human fetal femur–derived skeletal cells (FFDSCs), in 2-dimensional (2D) cocultures with HUVECs, displayed considerable modulation in the expression of osteogenic and angiogenic genes after supplementation with VEGF-165 (18). However, the modulatory effect of the osteogenic and angiogenic gene expression varied due to the rapid developmental plasticity of FFDSCs in cell culture monolayers. Although human skeletal fetal progenitor cells are a valuable source of information for the understanding of cell developmental processes and interactions, the application of FFDSCs in regenerative medicine is limited by ethical and practical limitations [reviewed by Gómez-Barrena *et al*. (19)].

Ferrera *et al.* (20) elegantly demonstrated the capability of 3D cell structures to enhance the continual differentiation process of osteoblasts toward an osteocyte phenotype by extending the culture period to 120 d. Cell monolayer sheets of osteoblasts formed 3D cell structures that were cultured submerged in osteogenic differentiation medium. Analysis of the 3D cell structures demonstrated an array of osteogenic proteins expressed, including collagen type I, osteopontin, osteonectin, bone sialoprotein, and fibronectin, after 25 and 48 d of culture. After 48 d of culture, osteocalcin was detected in cell structures, whereas alkaline phosphatase (ALP) was present in cells only at d 25 and 31 and not after 48 d. Furthermore, high levels of calcium incorporation were reported after 48 d of culture. Cellular structures were transplanted to a subcutaneous mouse dorsal model for a 20 d period, after which the cellular structures had formed an outer multilayered cellular collar rich in collagen matrix and a mineralized collagen rich core (20).

In a more recent study, chondrogenic priming of skeletal cells prior to spheroid formation was used by Freeman *et al*. (16) to observe changes in cell differentiation. Indeed, enhanced ALP expression and cartilage formation in coculture constructs of HUVECs/human bone marrow stromal cells (HBMSCs) after 14 d of culture were evident and were perceived as representing the initiation of endochondral bone formation. The chondrogenically primed HBMSCs were either cultured alone or cocultured with HUVECs (vascular priming). Regardless of HUVEC presence, cell priming produced sulfated GAG as well as an increase in ALP expression after 14 d of culture in contrast to unprimed and osteogenic-primed control groups. VEGF expression of the HBMSCs decreased upon the addition of HUVECs to the coculture, indicating that HUVECs were negating mesenchymal stem cell (MSC) signaling for vessel invasion (16). Furthermore, HUVECS were observed to attach to and infiltrate the cartilage template, and rudimentary vessels were formed in the HUVEC/MSC pellets after 1 wk of culture (16). Observations of neovascularisation in HUVEC/HBMSC cocultures were also reported by Goerke *et al.* (21). During cocultivation, HBMSCs were induced by HUVECs to differentiate into cells with a smooth muscle/pericyte phenotype (21). Goerke *et al.* (21) indicated that, in this setting, HUVECs increased smooth muscle actin expression in HBMSCs, mediated by direct cell contact and signaling *via* ERK, as opposed to a role for gap junction communication.

The current study investigated the potential of HUVEC/HBMSC coculture spheres to improve bone regeneration using an embryonic chick femoral defect model in organotypic culture over a 10 d period (**Fig. 1**). Sacchetti *et al.* (22) demonstrated that HBMSCs and HUVECs cotransplanted in Matrigel form capillary structures at 3 wk and more mature functional vessels at 8 wk.

**Figure 1:**
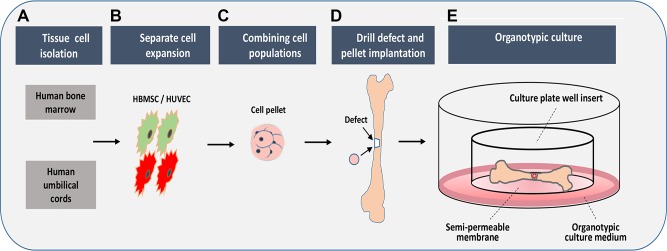
Overview of HUVEC/HBMSC pellet implants into chick femoral defects. *A*, *B*) Cells were isolated from human bone marrow and HUVECs and cultured/expanded separately in tissue culture flasks. *C*) Cells were pelleted either as monocell (HBMSC, HUVEC) or as combined cells (HBMSC+HUVEC), and the pellets were cultured for 2 d in conical tubes. *D*) Chick femurs were isolated, a drill defect was created mid-diaphysis, and the pellet was inserted into the defect. *E*) Femurs were maintained in organotypic culture for 10 d.

## MATERIALS AND METHODS

### Materials

Fertilized, wild-type D1, Bovans Brown chick eggs were obtained from Henry Stewart & Co. (Norfolk, United Kingdom). Tissue culture reagents were purchased from Lonza (Nottingham, United Kingdom). Fetal calf serum (FCS) was purchased from Thermo Fisher Scientific (Waltham, MA, USA). Endothelial cell growth supplement (ECGS) was purchased from Promocell (Heidelberg, Germany). Tissue culture plastics were obtained from Corning (Barry, United Kingdom). Cell culture plate inserts were procured from Thermo Fisher Scientific (Millicell-CM, 0.4 µm pore size, 30 mm diameter). Histologic reagents and materials were purchased from Thermo Fisher Scientific.

### Cell culture

Human bone marrow stromal cells (HBMSCs) were isolated and cultured from patients undergoing elective hip replacement surgery with ethical approval of the Southampton and South West Hampshire Local Research Ethics Committee (194/99/1). Cells were cultured at 37°C, 5% CO_2_/balanced air until confluent in basal minimal essential medium, Eagle α-modification (α-MEM) containing 10% FCS and 1% penicillin/streptomycin (P/S) (23). HBMSC cultures were expanded no higher than passage 3 in tissue culture flasks for all studies.

Human umbilical cords were obtained from consenting, healthy mothers at the Princess Anne Hospital, Southampton, United Kingdom, under ethical approval from Southampton and South West Hampshire Local Research Ethics Committee (LREC 05/Q1702/102). HUVECs were isolated and cultured as previously described (24) with minor modifications. Cells were dissociated from the vessel wall by infusion with 5 mg/ml (w/v) of Collagenase B solution (Roche Diagnostics, Burgess Hill, United Kingdom) and cultured at 37°C, 5% CO_2_/balanced air in endothelial cell culture medium consisting of medium 199 supplemented with 1% P/S, 10% FCS, ECGS/Heparin (ECGS/H) 0.4% (v/v) (Promocell).

### Organotypic bone defect cultures

#### Fluorescent cell labeling of HBMSC and HUVECs

Cell monolayers were harvested after trypsinization (Trypsin/EDTA). HBMSCs were incubated in 10 mM carboxyfluorescein diacetate (Vybrant Cell Tracer Kit V12883; Thermo Fisher Scientific) in 1 ml PBS for 15 min at 37°C. HBMSCs were repelleted and resuspended in culture medium. The cell suspension was incubated for 30 min at 37°C, and cells were washed twice prior to transfer into 15 ml conical tubes. HUVECs were incubated in 5 µl/ml of 10^6^ cells in Vybrant Dil cell labeling solution (V22885; Thermo Fisher Scientific) for 20 min at 37°C, repeatedly washed, and resuspended in culture medium.

#### Egg incubation

On arrival (d 0), chick eggs (*Gallus domesticus*) (fertilized, d 1) were incubated in a humidified Hatchmaster (Brinsea, Weston-super-Mare, United Kingdom) at 37°C for 18 d with continual 90° rotation.

#### 3D coculture spherical constructs of HUVECs and HBMSCs

HBMSCs and HUVECs were cultured, washed in PBS, and incubated with Trypsin/EDTA at 80–90% confluency. For monocell pellets, a cell density of 2.5 × 10^5^ of HUVECs and HBMSCs was used. For coculture pellet constructs, a 1:1 ratio of HUVECs/HBMSCs (1.25 × 10^5^ cells each) was used. Cells were pelleted by centrifugation in a 15 ml conical tube at 1000 rpm for 5 min. The cell pellets were incubated in a 50/50 mixture of endothelial cell medium and α-MEM supplemented with 1% P/S without FCS at 37°C, 5% CO_2_/balanced air under humidified conditions for 48 h. Monopellets were simultaneously cultured in their respective media: HBMSCs (α-MEM, 1% P/S) and HUVECs (Medium 199, 1% P/S, 0.4% ECGS) without fetal calf serum supplementation.

#### Embryonic chick femora dissection, drill defect application, and pellet implantation

**Figure 2:**
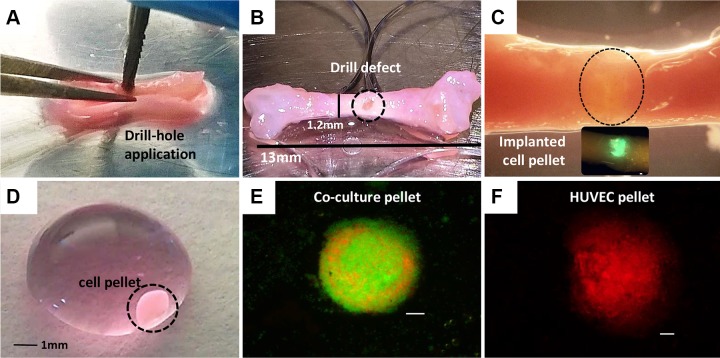
Drill defect application and pellet implantation in E18 chick femurs. Representative images of the preparation of E18 chick femur drill defects implanted with cell pellets for a 10 d organ culture. *A*) Creation of drill defect using a sterile 0.9 mm metal drill bit. *B*) Femur with applied drill defect (circle). *C*) Femur with pellet implanted into the drill defect (circle) prior to the 10 d organotypic culture period. *D*) Cell pellet suspended in medium in a Petri dish prior to implantation. *E*, *F*) Sample images of fluorescently labeled coculture cell spheres HBMSC/HUVEC (green, Vybrant carboxyfluorescein diacetate; red, Vybrant Dil) and HUVEC only (Vybrant Dil), at d 8 of *in vitro* culture. Scale bars, 100 µm.

On d 18 of incubation, the embryos were removed, transferred to glass Petri dishes, and immediately culled by decapitation. Femurs were dissected, and soft tissue was removed to expose the bone. A 0.5 mm metal pin was used to mark an initial drill hole in the center of the diaphysis, and a 0.9 mm drill bit was used to drill and generate the defect (**Fig. 2*A*, *B***). Cell pellet constructs (Fig. 2*D*) were carefully aspirated from the 15 ml tubes using a 1 ml pipette tip, and the individual cell construct was transferred to the bone drill defect created in the chick femurs (Fig. 2*C*). Forceps were used to carefully place the construct into the bone defect.

#### Organotypic culture

Four femurs were prepared for each treatment group (HUVEC pellets, HBMSC monocell pellets, and HUVEC/HBMSC coculture pellets). A no-pellet control group without cell pellet construct was added. Femurs were transferred to an organotypic culture well insert with a 0.4 µm pore size, 30 mm diameter membrane on which the samples were placed. Samples were imaged and cultured at the air/liquid interface of the insert with 2 femurs per insert placed into a 6 well plate containing 1 ml of organotypic culture medium (α-MEM, 1% P/S, supplemented with 2 µl/ml ascorbic-2-phosphate) (MilliporeSigma, Dorset, United Kingdom). For sham controls, 4 femurs containing drill defects without a pellet construct added were cultured simultaneously. The femurs were cultured for 10 d in a 5% CO_2_/balanced air incubator with medium changes performed daily. The organotypic cultured femurs were harvested on d 10 and imaged prior to fixing in 4% paraformaldehyde.

### Microcomputed tomography

For quantitative 3D analysis, chick femurs were scanned pre- and post culture using a SkyScan 1176 micro-computed tomography (µCT) scanner (Bruker, Kontich, Belgium) under the following settings: X-ray source 40 kV, 600 µA, 496 ms exposure time, voxel size 35 µm. Femurs were securely held in a 0.5 ml sterile Eppendorf tube. Raw data were reconstructed using NRecon software v.1.6.10.4, correcting for beam hardening (30%), ring artifacts, and misalignment. CTAn software v.1.16 was used to visualize and analyze the reconstructed images for bone volume (BV) and BV/tissue volume (TV) ratio. For the analysis, a volume of interest consisting of 50 cross-sections over the region of interest (25 transverse cross-sections) from either side of the center of the defect, including some of the peripheral bone tissue, was selected by the CTAn software; this was undertaken for each femur at d 0 and d 10. Otsu thresholding was initially applied to obtain an average binarized lower and upper gray scale threshold for the reconstructed datasets (25). Scans performed at d 0 and d 10 were calibrated to a calcium hydroxyapatite control phantom (Bruker) that was scanned with the samples. The resulting data were normalized to d 0. To visualize the femurs, a 3D model was created using CTVox software v.3.2.0. All software used for µCT was provided by SkyScan (Bruker).

### Tissue processing and sectioning

After µCT analysis, femur samples were fixed in 4% paraformaldehyde-PBS overnight at 4°C and dehydrated through increasing ethanol concentrations (50, 90, and 100%). The tissue was cleared in Histo-Clear (National Diagnostic, Atlanta, GA, USA) prior to immersion in liquid paraffin wax at 60°C to ensure full penetration by the wax. The samples were embedded in paraffin blocks for sectioning. Sections were cut at 7 µm on a microtome (Microm 330; Optec, Oxfordshire, United Kingdom) and transferred to preheated glass slides at 37°C for ∼2 h.

### Alcian Blue/Sirius Red staining

Tissue slide sections were brought to room temperature and rehydrated through Histo-Clear and a series of ethanol solutions (100, 90, and 50%). Weigert’s hematoxylin (MilliporeSigma) was applied to the slide sections to stain the cell nuclei. Slides were immersed in 0.5% Alcian Blue 8GX in 1% acetic acid to stain for proteoglycans. Slides were treated in 1% molybdophosphoric acid (MilliporeSigma) prior to staining with 1% Sirius Red F3B (Direct Red 80; MilliporeSigma) for collagen. Slides were washed in H_2_O, dehydrated in increasing concentrations of ethanol (50, 90, and 100%), and cleared in Histo-Clear before mounting with dibutyl phthalate xylene.

### Immunohistochemical analysis

Exogenous peroxidase was quenched using 3% H_2_O_2_ (MilliporeSigma), and 1% bovine serum albumin in PBS was applied. The sections were incubated overnight at 4°C with primary antibody, followed by anti-rabbit IgG (biotinylated) (1:100; Dako, Santa Clara, CA, USA). To visualize the immune complex, Extravidin Peroxidase (MilliporeSigma) was applied, followed by incubation with 3-amino-9-ethylcarbazole in acetate buffer with 0.015% H_2_O_2_, resulting in red/brown staining. Sections were counterstained with Alcian Blue and light green and mounted with hydro-mount. Primary antibodies (rabbit polyclonal) were obtained as follows: type I collagen (COL I, LF68, 1:1000) (kindly donated by Dr. Larry Fisher, National Institutes of Health, Bethesda, MD, USA), CD31 (PECAM-1, 1:100; Proteintech, Manchester, United Kingdom), type II collagen (COL-II, 1:500; Calbiochem, Merck-Millipore, Watford, United Kingdom), and von Willebrand factor (vWF) (1:100; DakoCytomation, Cambridgeshire, United Kingdom).

### Image capture

Images of slide and tissue samples were captured and processed using a Zeiss Axiovert 200 inverted microscope with Axiovision software (version 4.7). Confocal imaging was performed using a confocal laser scanning platform (TCS SP8; Leica Microsystems, Milton Keynes, United Kingdom) and processed using Leica Application Suite X, software version 2.0.0.14332.

### Statistical analysis

Data derived from at least 4 replicates from 3 experiments were analyzed using 1-way ANOVA with Dunnett’s multiple comparison *post hoc* test using GraphPad Prism 6 software (v.6.07; GraphPad Software, La Jolla, CA, USA). The resulting data for BV and BV/TV of each femur were normalized to the corresponding D0 scan data to obtain changes in BV (ml^3^) and BV/TV (%) over the culture period.

## RESULTS

### Confocal microscopy analysis of the spatial regions of skeletal and endothelial cell occupation in 3D pellets

**Figure 3:**
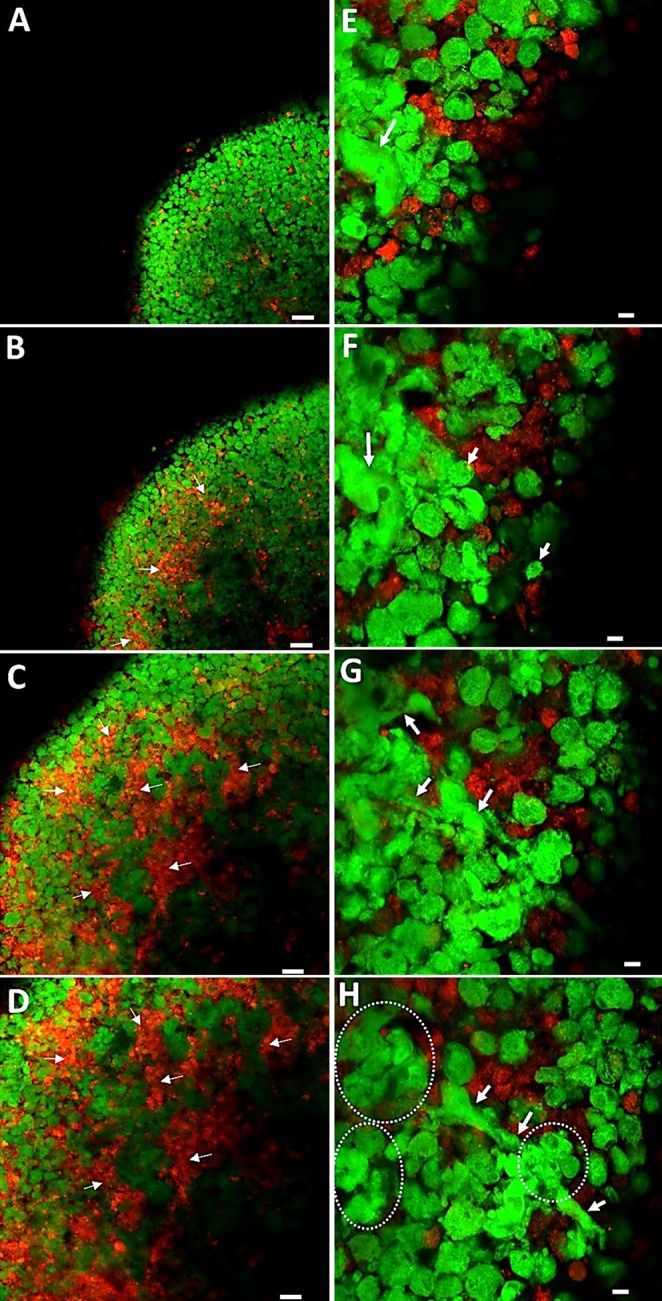
Confocal microscopy images. *A*–*D*) Visualization of consecutive layers through a d 8 coculture cell pellet construct of HBMSCs (Vybrant, green) and HUVECs (Vybrant Dil, red). Snapshot images throughout the cell pellet constructs from the outside (*A*, *E*) to the inside (*D*, *H*). HBMSCs were observed predominantly in the outer layer of the pellet (*A*), with HUVECs increasing in number toward the center of the pellet (*B*–*D*). Arrows depict areas of HUVEC aggregation. *E*–*H*) Higher-magnification images depicting cell positions and migration within consecutive layers of the cell pellet construct. *E*, *F*) Visible variations in morphology of HBMSCs (arrows). *G*, *H*) Elongation of HBMSCs (arrows). *H*) Clusters of HBMSC aggregates (dotted lines). Scale bars, 50 µm (*A*–*D*); 10 µm (*E*–*H*).

Fluorescently tagged cell types were clearly distinguishable throughout the coculture sphere (Figs. 2*E*, *F* and **3**), with HUVECs (red) and HBMSCs (green) displaying distinct regional areas of location within the sphere. The coculture pellets of HUVECs/HBMSCs and HUVECs displayed a tighter spherical, compact composition. Confocal microscopy sequential images showed vivid cell activity of HBMSCs and HUVECs, with the HBMSCs in particular displaying migratory activity evident in the elongation of cells across other cells, the cluster formation of cells, and general heterogeneity in cell size and morphology (Fig. 3). HBMSCs were abundant at the outer layer of the sphere (Fig. 3*A*). In contrast, HUVECs resided predominantly within the increasing depth of the sphere (Fig. 3*B–D*). Although a modest degree of intermixing of each cell type was observed toward the center of the pellets (Fig. 3*C*, *D*), HUVECs and HBMSCs resided largely within distinct compact clusters (Fig. 3*D*, arrows). High magnification confocal microscopy revealed the HBMSCs extending in length (Fig. 3*E–H*, arrows) to neighboring HBMSCs and forming dense cell congregates (Fig. 3*D*, circles), whereas HUVECs remained clustered in niches between the cell layers. The HBMSCs displayed a more heterogeneous size distribution compared with the HUVEC population.

### µCT analysis of bone formation in femur defects with added cellular pellets

**Figure 4:**
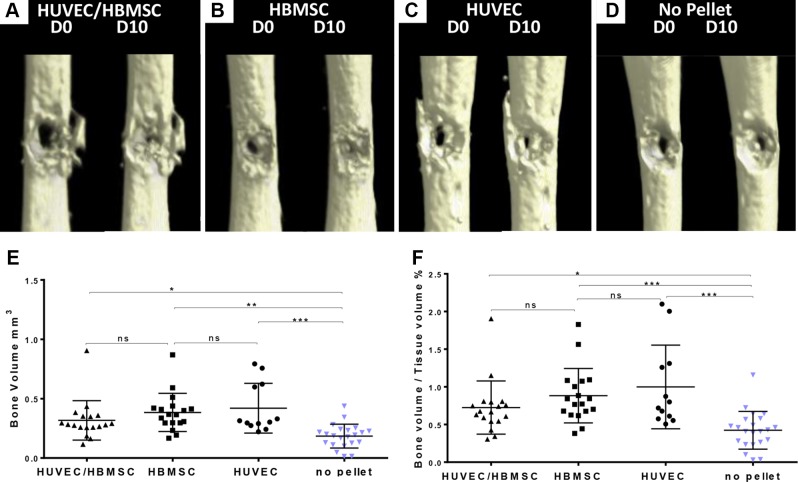
µCT scan images of representative femur defects at d 0 and 10, and statistical analysis. *A*) Coculture pellet (HUVECs/HBMSCs). *B*) HBMSC pellet. *C*) HUVEC pellet. *D*) No pellet. *E*, *F*) µCT data analysis of increase in BV (*E*) and BV/TV ratio (*F*) change at d 10 in femur defects with cell pellet implant compared with sham control. All treatment groups demonstrated a significant change in bone parameters. No-pellet femurs constituted sham controls. HUVEC pellets, *n* = 12; HBMSC pellets, *n* = 18; HUVEC/HBMSC pellets, *n* = 18; no pellet, *n* = 22. All data presented as mean ± sd. *P ≤ 0.05, **P ≤ 0.01, ***P ≤ 0.001.

µCT analysis demonstrated enhanced closure of defects at d 10 in the cell construct–implanted femurs compared with empty sham controls. Comparative µCT images of femurs at d 0 and 10 of organ culture of each treatment demonstrated a distinct visible reduction in the d 10 femur drill defects compared with d 0, in particular in femur samples implanted with cell pellets (**Fig. 4*A–D***). Some bone fragment remnants from the drill-defect application were visible around the defect area on the µCT images (Fig. 4*A*, *C*).

A statistically significant increase in BV and BV/TV at d 10 was demonstrated for all treatment groups, with the HUVEC (BV, *P* < 0.0002; *n* = 12) and HBMSC (BV, *P* < 0.0004; *n* = 18) pellet treatment groups demonstrating the highest increase in BV/TV (Fig. 4*E*, *F*) compared with the no-pellet control femurs (*n* = 22). Reduced relative numbers were due to some cell pellets not forming properly. Any additional femurs with drill defect and without attempted pellet implantation were included in the no-pellet control group. Overall values obtained for BV of the coculture pellet–implanted femurs were slightly lower relative to the monocell construct–implanted femurs (BV, *P* < 0.0250; *P* = 18). The results from the µCT data analysis were reflected in the comparative µCT images of Fig. 4, with the most significant changes in bone-forming parameters evident in the HUVEC pellet implants compared with all other treatment groups.

### Integration of 3D coculture constructs within the drill defect of embryonic chick femurs

**Figure 5:**
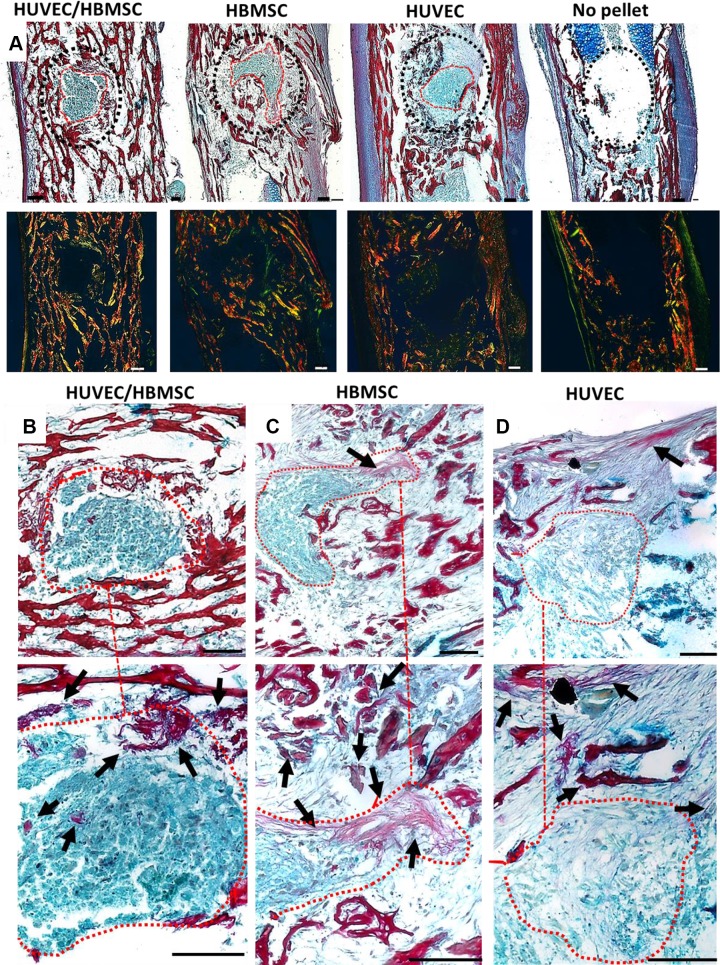
Representative histology images depicting cell pellet construct integration (circled red) within femoral defects stained with Alcian Blue (proteoglycans)/Sirius Red (collagen). *A*) Overview of pellet integration in all treatments and the no-pellet control sample. A dotted black line surrounds the defect area. Images underneath depict birefringence imaging of collagen fiber distribution (thick collagen fibers shown in orange-red; thin collagen fibers shown in green). *B*–*D*) Magnified images of the pellet implant of each treatment group; higher-magnification images of the same area below (dotted line). Femur drill defects containing remnants of HUVEC/HBMSC coculture pellet (*B*), HBMSC pellet (*C*), and HUVEC pellet (*D*). Arrows show collagen formation within and around the defect area. Scale bar, 100 µm.

Cell pellets implanted within chick femoral defects displayed strong integration with the surrounding bone tissue in the majority of the different cell groups. In contrast, negligible changes were observed in the sham (no-pellet) control femurs at d 10 (**Fig. 5*A***). Increased collagen staining was present throughout the bone tissue of the pellet-implanted femurs compared with the controls. This was confirmed by birefringence imaging of collagen fibers stained with Sirius Red within the defect. Thin green fibers, representing less mature fibers, were visible in and around the defect area, near the remnants of the pellet, whereas red-orange fibers were predominantly visible throughout the existing bone trabeculae. Moreover, a marked thickening of the perichondrium adjacent to the defective area of the femur containing implanted HUVEC pellets was noted.

There were variations between treatments in the degree of pellet retention *in situ*, and collagen strands could be seen extending to or from the pellet and the surrounding tissue (Fig. 5*C*, *D*); these were particularly evident between the coculture and the HUVEC pellet defects. Throughout the coculture pellet implants, a dense, compact region of collagen was observed (arrows). Collagen-stained fibers extended from HBMSC pellet implants to the adjacent marrow tissue (Fig. 5*C*). There was a less defined presence of collagen within the HUVEC-implanted defects (Fig. 5*D*), with a more fragmented appearance of collagen fibers (arrows).

### Characterization of collagen expression and bone mineralization within femur defects with implanted cell constructs

**Figure 6:**
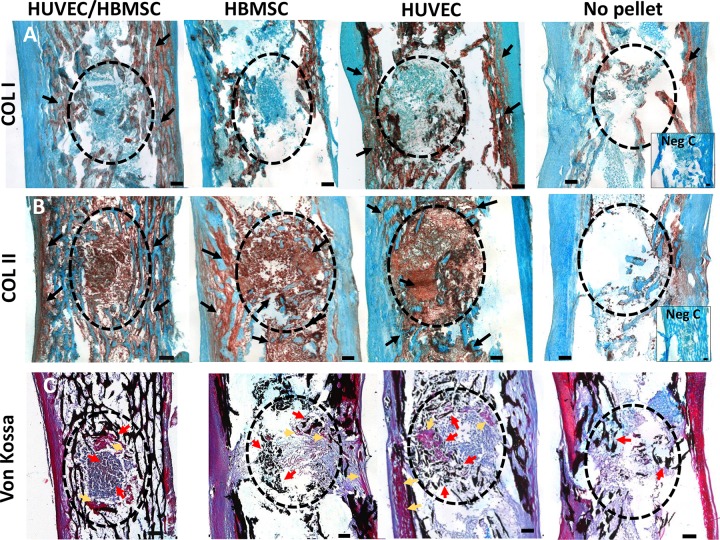
Representative tissue sections of type I collagen (*A*), type II collagen (*B*), and von Kossa mineral staining (*C*) in E18 chick femur defects with and without pellet implant. *A*) COL I (type I collagen) within bone trabeculae (arrows) but not within pellet defects (circles) in all treatment groups. *B*) Enhanced COL II (type II collagen) presence in all cell pellet implants and surrounding bone tissue (arrows) compared with no-pellet controls. *C*) von Kossa staining showing black mineral deposits within the defects (red arrows), particularly in HBMSC and HUVEC pellet–implanted femurs. Pockets of osteoid matrix (pink/purple) were observed adjacent to coculture and HUVEC pellets (yellow arrows). Negative controls for immunostaining (Neg C) had primary antibody omitted. Scale bar, 100 µm.

In the control chick femurs, the level of expression of localized type I and II collagen staining was overall weak compared with the implanted pellet groups. Intense type I collagen staining was observed in the bone trabeculae of pellet-implanted femurs, although there was no evidence of type I collagen staining within the pellet implant (**Fig. 6*A***). In contrast, type II collagen staining was present within defects of coculture and monocell pellet groups as well as within the trabeculae region surrounding the defect (Fig. 6*B*).

There was evidence of calcium deposition, confirmed by von Kossa staining, in all pellet-implanted femurs compared with no-pellet controls. However, overall negligible von Kossa staining was observed within the pellets (Fig. 6*C*). Enhanced calcification was visible within the trabeculae regions of the diaphysis. There was clear evidence of calcium deposits within and around the defects implanted with HUVEC and HBMSC pellets, compared with no-pellet controls, with concentric accumulation around the pellets intermixed with osteoid formation (pink/purple, arrows), which in part were remnants of pre-existing bone.

### Expression of angiogenic proteins in the cell construct–implanted femurs

**Figure 7:**
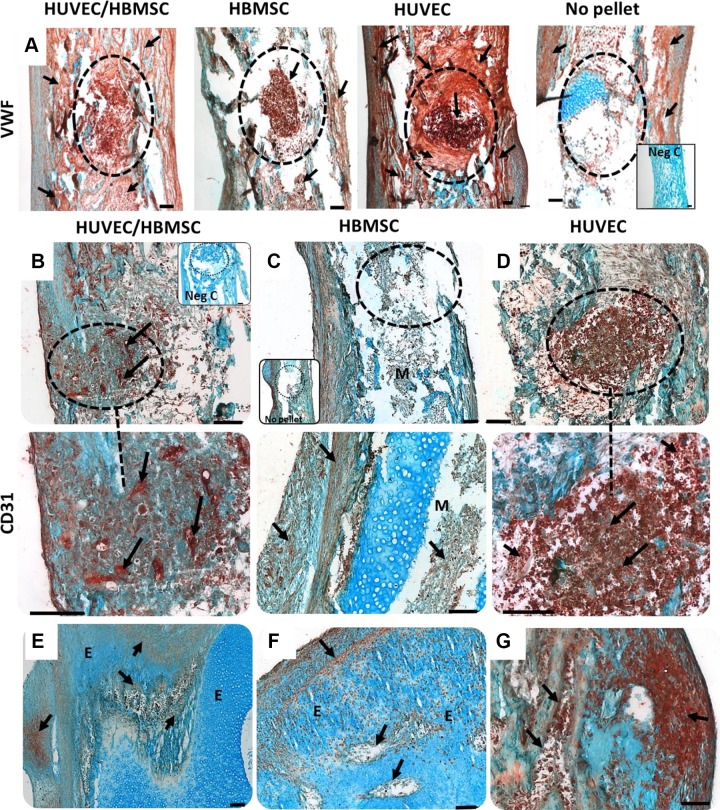
*A*–*F*) vWF and CD31 (PECAM-1) immune expression in mono- and coculture pellet/defects. *A*) Representative images of vWF antigen were enhanced in all pellet treatments, particularly in implants containing HUVEC fractions (visible gaps in surrounding tissue at marrow cavity) as well as the surrounding tissue (arrows). *B*–*G*) Immunohistochemistry for CD31 expression in E18 chick femoral drill defects depicting different areas of 1 femur for each treatment. *B*–*D*) HUVEC/HBMSC coculture pellets (*B*), BMSC pellets (*C*), and HUVEC pellets (*D*); negative control (Neg C), where the primary antibody was omitted (inset). *B*, *D*) Dotted circle depicts defect area and below at higher magnification, with *B* showing distinct CD31 stained aggregates within the pellet (arrows). *E*–*G*) CD31-positive staining within the epiphysis of femurs. Note the tissue (*G*) infiltrated with CD31-positive cells along the periosteum of the femur. Arrows depict areas of immune-positive staining. E, epiphysis; M, marrow cavity. Scale bars, 100 µm.

vWF expression was observed throughout the pellets and bone defect areas, with staining radiating into the diaphysis (**Fig. 7*A***). Intense vWF staining was evident within the implanted HUVEC pellets expanding in a concentric pattern of vWF staining beyond the implanted HUVEC pellet defect area into the marrow cavity. The coculture femurs also displayed enhanced vWF expression within the pellet region and the remaining femur, but the expression was less intense and more evenly distributed compared with that of the HUVEC pellet group. HBMSC pellets displayed strong expression of vWF within the pellet, although the tissue adjacent to the defect was modestly stained.

Expression of CD31 was most evident in the HUVEC pellet–implanted defect femurs (Fig. 7*D*, arrows) and was distinctly visible within the pellet, similar to the vWF immunodetection. A dense accumulation of CD31-positive cells was noted in the periosteum adjacent to the HUVEC pellet–implanted defect (Fig. 7*D*). Additionally, CD31-positive cell clusters were present in areas surrounding the HUVEC-implanted femoral defect. In coculture cell pellets, elongated areas of CD31-positive stained cells were clearly observed (Fig. 7*B*, arrows), indicating endothelial cell aggregates within the pellet. In the epiphysis apex of the femurs, CD31-positive cells (Fig. 7*B*, *C*) were evident proximal to emerging growth plate–like channels (Fig. 7*B*). The HBMSC pellet group displayed negligible CD31 expression within the defect area (Fig. 7*C*), unlike the staining pattern observed of vWF antigen within the HBMSC pellet and compared with the HUVEC and coculture treatment groups. HBMSC femurs showed limited CD31 expression throughout.

## DISCUSSION

The current study has revealed significant bone formation at the defect site of chick femora implanted with co- and monocell pellets of HBMSCs and HUVECs used to bridge and repair the defect. Parameters for bone formation were significantly higher in all 3 treatment groups, but, contrary to expectations, the coculture pellet model did not produce the largest increase in BV and BV/TV compared with the monocell pellets. The most significant increase in bone-forming parameters was observed in defects implanted with HUVEC and HBMSC monoculture pellets (*P* < 0.001) compared with sham controls. The current studies used previously acquired knowledge from 2D coculture studies (18) of human skeletal and endothelial cells and enhanced stimulation of osteogenic markers ALP and type I collagen while recognizing the typical limitation of a 2D coculture and the absence of spatial dynamics and cell motility.

The current study has demonstrated enhanced bone formation through the creation of a niche of cell-only graft in an *ex vivo* femoral defect without the use of external supplements. This unique model of 3D organ culture supersedes even the most complex *in vitro* experiments that fail to demonstrate the complex interaction with indigenous tissue and precedes *in vivo* testing by merely mimicking the *in vivo* conditions, thus using a seamless technique with the potential to reduce preclinical animal testing.

The use of HBMSC pellets has previously been reported to enhance osteogenic differentiation, with evidence of increased mineralization and improved bone regeneration (9). Equally enhanced bone regeneration was reported after the application of chondrocyte differentiated pellets grafted onto rat nonunion defects, with the aim to induce endochondral ossification. The pellets showed evidence of mineralization and the presence of hypertrophic chondrocytes after 7 and 14 d of implantation (26). However, no previous study has shown a superior enhanced bone repair with the use of HUVEC monocell pellets compared with skeletal cell or coculture cell pellets.

We hypothesize that the implanted cell pellets exchange signals to and from cells residing in the bone tissue to induce recruitment of cells and blood vessels for repair. The presence of 2 major cell types (endothelial cells and HBMSCs) within the coculture pellets was expected to create a unique niche and produce an enhanced impact on bone healing. Although positive results were observed, the slightly lower levels of bone formation of the coculture pellets could have been be due to the cells being at different stages of differentiation within the pellet construct after initial *in vitro* culture. It is possible that combining the endothelial and osteoprogenitor bone cell types triggered an increase in matrix production, accounting for a tighter bond between the cell types, rendering the cells less mobile and reducing the capacity to signal and interact with the host tissue.

 It cannot be dismissed that the cells within the pellet may have self-organized and that cell signaling was initially more dynamic between cells within pellets than within the host tissue. Indeed, researchers have previously reported proliferation and self-organization of HUVECs within coculture spheres and increased mineralized matrices in stand-alone *in vitro* hydrogel bioprinted constructs (27). In our studies, the staining profile of CD31 indicated increased endothelial migratory activity, in particular in femurs implanted with HUVEC pellets. Alcian Blue/Sirius Red staining confirmed an increased cell-mobile structure of both monocell pellets in contrast to the more distinct and compact coculture pellets. Overall, the HUVEC pellets with a heterogeneous appearance were harder to distinguish from the surrounding tissue, indicating a cell-cell interactive process within the implant/defect niche. In particular, vWF and CD31 protein expression was strongly associated around the defect with HUVEC pellets compared with all other treatments. These results indicate that HUVECs within the pellet trigger the release of factors such as vWF either directly or *via* signaling molecules onto the surrounding cells or tissue. Von Kossa staining confirmed calcium deposition within and adjacent to the defect area in both the HUVEC- and HBMSC-implanted pellets compared with sham controls. In contrast, calcification in the coculture pellet femurs was less abundant at the original implant site.

These findings suggest that the HUVECs and HBMSCs play an important role in the migration into (and potentially recruitment of cells from) the surrounding regions of the defect area. Indeed, endothelial subtypes have previously been identified to be involved in the coupling of angio- and osteogenesis (28); furthermore, Langen *et al.* (29) have recently associated specific endothelial types associated with osteoblast lineage support during development and osteogenesis.

We hypothesize that, within a coculture pellet, both cell types interact primarily with each other, which is evident in the dense plug formation and the lack of migration observed within the coculture pellet defects. One reason for this could be the close proximity of the 2 cell types (10) and that biochemical exchange and matrix formation is localized. Thus, more cell resources were expended by cell-to-cell communication within pellets rather than through extended communication with the surrounding cells. It has previously been shown that the number of interendothelial cell junctional complexes is thought to increase significantly in cocultured spheroid constructs of endothelial and smooth muscle cells, visible at endothelial cell junctions (30). Similarly, HUVECs in contact with HBMSCs within the pellet may produce increased intercellular junctions, accounting for the compact pellet structure of the pellet. However, it would be interesting to observe if and how these interactions change over an extended culture period.

The current study revealed CD31-positive staining of small, elongated cell aggregates within the coculture pellets. Similarly, in a HUVEC/MSC spherical pellet coculture (1:1 ratio) over a 7 d period, Saleh *et al.* (31) observed self-assembly of the cells into organized structures of cell-specific segregation within the sphere. This resulted in distinct CD31-positive islands within the pellets, and over time the HUVECs formed substantial networks within the structure. However, HUVECs also inhibited proliferation of MSCs in the coculture pellets, reinforcing the findings of the current study that coculture pellets remained distinctly within the defect region. It is possible that the distinct microenvironment of the coculture pellet has the potential to encourage the phenotypic differentiation of the endothelial cells present toward a particular subtype contributing to the spatial morphology of the coculture pellets, in contrast to the individual HUVEC and HBMSC pellets. A subtype of endothelial cells has previously been identified in murine bone that is situated in specific niches of the bone (metaphysis and endosteum) that express high levels of CD31 and endomucin (28). This subtype (H) of endothelial cell has a strong spatial association with osteoprogenitor cells and is said to mediate bone vasculature growth, establishing a phenotypic heterogeneity in the long bone endothelium (28).

Conversely, the findings of the current study suggest that the coculture conditions within the pellet construct could have produced an effect similar to priming of the HBMSCs (16, 26, 32, 33). Immunohistochemical findings indicated enhanced vWF and CD31 expression, indicating angiogenic activity within cocultures as well as the presence of COL-II. This indicates the possibility of cell interaction within cocultured pellets, encouraging endochondral bone healing, using an intermediate cartilaginous step during wound healing prior to vascularization. Freeman *et al*. (16) demonstrated cartilage formation of chondrogenic primed HBMSC pellets. Subsequent coculture with HUVECs produced CD31-positive cells attaching to the pellet periphery and infiltrating the cartilaginous template after 1 wk. Only when HUVECs and HBMSCs together were added to the chondrogenic pellet (not HUVECs alone) was rudimentary vessel formation observed.

Although the current study implemented a cell-only graft, the results confirmed the potential and importance of indirect cell priming by the local niche environment and, critically, vascular priming and modulation by the presence of HUVECs. The implanted cell pellets in this model are embedded in a heterogeneous bed of dynamic bone cells, creating a local niche environment and priming cells to migrate and proliferate from a mix of temporal signals between implant and defect. Thus, an increased presence of vWF in the pellets and bone defect tissue containing the HUVEC fractions is a possible response to stimuli originating from the drill injury and subsequent changes in pH level. Various cell types, including fibroblasts (34) and osteoblasts (35), are able to secrete vWF into the cytoplasm by constitutive secretory pathways (34), accounting for the presence of vWF in all treatment groups.

This study has demonstrated that pellets of HUVECs and HBMSCs and cocultures of both cell types can enhance bone formation at a bone defect site and increase the expression of osteogenic and angiogenic markers. Results from these studies elucidate an important role of endothelial cells (alone) in this *ex vivo* defect model and indicate that the differential in bone forming parameters with the coculture constructs may arise as a consequence of delayed interactive construct response within the tissue caused by the distinct compact morphology of the pellets with less initial migratory cell activity. A less compressed pellet design (10, 36, 37) and a longer culture period of this model would be useful to examine this theory. The results from the current study highlight the efficacy of this model as an inexpensive, high-throughput *ex vivo* model for preliminary research.

In summary, pellets of HUVECs and HBMSCs and cocultures of both cell types enhance bone formation at a bone defect site and demonstrate the importance of the osteogenic-endothelial niche interaction in bone regeneration. Elucidation of the component cell interactions in the osteogenic-vascular niche in driving the osteogenic processes observed in this defect model will aid translation of bone reparative strategies with significant therapeutic implications therein.

## Abbreviations

2D: 2-dimensional
3D: 3-dimensional
α-MEM: minimal essential medium, Eagle α-modification
μCT: micro-computed tomography
ALP: alkaline phosphatase
BV: bone volume
ECGS: endothelial cell growth supplement
FCS: fetal calf serum
FFDSC: fetal femur-derived stem cell
HBMSC: human bone marrow stromal cell
MSC: mesenchymal stem cell
P/S: penicillin/streptomycin
TV: tissue volume
vWF: von Willebrand factor
